# *pyBioPortal*: a Python package for simplifying cBioPortal data access in cancer research

**DOI:** 10.1093/jamiaopen/ooae146

**Published:** 2024-12-26

**Authors:** Matteo Valerio, Alessandro Inno, Stefania Gori

**Affiliations:** Medical Oncology, IRCCS Sacro Cuore Don Calabria Hospital, 37024 Negrar di Valpolicella, Verona, Italy; Medical Oncology, IRCCS Sacro Cuore Don Calabria Hospital, 37024 Negrar di Valpolicella, Verona, Italy; Medical Oncology, IRCCS Sacro Cuore Don Calabria Hospital, 37024 Negrar di Valpolicella, Verona, Italy

**Keywords:** cBioPortal, cancer research, bioinformatics, Python

## Abstract

**Objectives:**

In recent years, the rise of big data and artificial intelligence has led to an increasing expansion of databases and web services in biomedical research. cBioPortal is one of the most widely used platforms for accessing cancer genomic and clinical data. The primary objective of this study was to develop a tool that simplifies programmatic interaction with cBioPortal’s web service.

**Materials and Methods:**

We developed the *pyBioPortal* Python package, which leverages the cBioPortal REST API to access genomic and clinical data. The retrieved data is returned as a Pandas DataFrame, a format widely used for data analysis in Python.

**Results:**

*pyBioPortal* offers an efficient interface between the user and the cBioPortal database. The data is provided in formats conducive to further analysis and visualization, promoting workflows and improving reproducibility.

**Discussion:**

The development of *pyBioPortal* addresses the challenge of accessing and processing large volumes of biomedical data. By simplifying the interaction with the cBioPortal API and providing data in Pandas DataFrame format, *pyBioPortal* allows users to focus more on the analytical aspects rather than data extraction.

**Conclusion:**

This tool facilitates the retrieval of heterogeneous biological and clinical data in a standardized format, making it more accessible for analysis and enhancing the reproducibility of results in cancer informatics. Distributed as an open-source project, *pyBioPortal* is available to the broader bioinformatics community, promoting collaboration and advancing research in cancer genomics.

## Introduction

In recent years, with the advent of big data and artificial intelligence, there has been a growing proliferation of databases and web services in the field of biomedical research,^1^ which have now become essential tools for sharing and accessing crucial information in cancer informatics. Data access is fundamental in cancer research, where the analysis of large amounts of genomic, transcriptomic, and clinical data can lead to new discoveries and therapeutic developments.cBioPortal^2–4^ is one of the most widely used platforms for accessing such data, offering both an interactive web-based exploration interface and public Representational State Transfer (REST) Application Programming Interface (API)^5^ for retrieving information from a wide range of cancer studies. However, directly using the downloadable data from cBioPortal without significant processing, or specifically its available API, could be challenging for researchers and university students without specific programming skills, as the data may be presented in complex, nested formats that require conversion into usable tables. Moreover, users who prefer to focus on data analysis and interpretation rather than on the technical aspects of data extraction and preparation may find these tasks prohibitive. To address this issue, a new Python package called *pyBioPortal* has been developed.

## Objectives

The main objective that led to the development of *pyBioPortal* is to simplify programmatic interaction with cBioPortal’s public API and provide the retrieved data in a standardized format, directly usable for analysis, with the aim of improving the reproducibility of results in cancer research.

## Materials and methods

### Implementation and architecture

Python 3^6^ was chosen as the programming language for *pyBioPortal*, given its growing popularity in bioinformatics and compatibility with all major operating systems. The version of Python used for development is 3.9, which ensures stability and support in the more recent versions of Python, as version 2 has not been supported since 2020.

**Figure 1.**
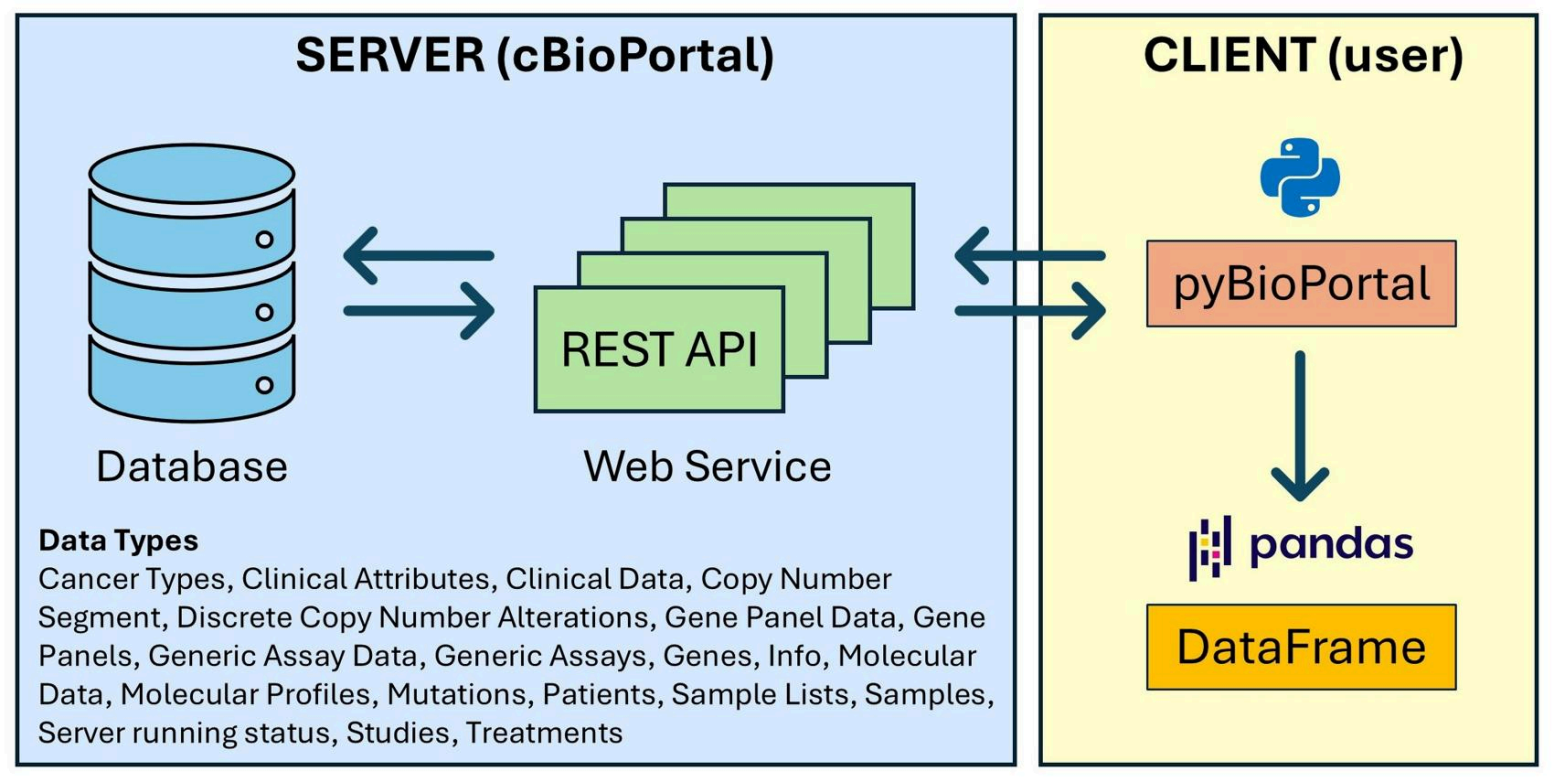
Schematic representation of the data flow from cBioPortal to the user through *pyBioPortal*.

The package uses the REST API web services provided by cBioPortal to retrieve the data of interest, leveraging external libraries such as Requests^7^ and Pandas^8^ for handling Hypertext Transfer Protocol (HTTP) requests^9^ and processing the retrieved data. The acquired and appropriately processed data are returned to the user as a Pandas DataFrame object, a widely used format for data analysis in Python, as shown in Figure 1.

The structure of the package is organized into modules, each of which is dedicated to a specific type of data, as defined by the cBioPortal API described in the related documentation (https://www.cbioportal.org/api/swagger-ui/index.html) and shown in Figure 1.

Each module in the package consists of various functions that provide simplified access to the resources, handling the interaction with the web service transparently for the user, without the need to know how the HTTP request to be sent to the server should be structured.

*pyBioPortal* manages the entire set of endpoints provided by the cBioPortal REST API, supporting HTTP requests with both GET and POST methods. The functions within each module are named according to a convention outlined in the API documentation, which identifies the type of request sent to the server: functions using the GET method are prefixed with “get,” while those using the POST method are prefixed with “fetch.”

### Database query and data retrieval

To specify and request which data you want to retrieve from the cBioPortal database, it is necessary to define a query having parameters whose type and meaning are described in the online documentation provided by cBioPortal. For this purpose, by properly setting these parameters and passing them as arguments to the relevant *pyBioPortal* functions, based on the type of data of interest, the following operations are performed transparently for the user: composing the HTTP request to send to the server; parsing the responses obtained from the web API provided in JSON format (JavaScript Object Notation)^10^; processing the results and returning a Pandas DataFrame containing the desired data.

To deliver data directly in a tabular format as a Pandas DataFrame, the package leverages private auxiliary functions, like *flatten_dict_columns* and *flatten_dict_list_columns*, to automatically convert nested dictionaries and lists of dictionaries into flat DataFrame columns. This internal processing is entirely transparent to the user, allowing for immediate access to the data in a usable, analysis-ready format without requiring additional data manipulation. This feature exemplifies the package’s approach to simplifying data extraction from complex JSON formats (for technical details, full function implementations are available in the GitHub repository).

In addition to the standard parameters required by the API, some functions accept additional arguments that allow for more processed results. For example, it is possible to obtain a DataFrame with data organized in either “wide” or “long” format. In the “wide” format, variables associated with the same record (ie, associated with the same patient or sample) are arranged across multiple columns, which can be useful for visualizations or specific types of statistical analyses (an illustrated example of this transformation is provided further in the text). Conversely, in the “long” format, these variables are spread over multiple rows, allowing for easier application of certain data manipulation techniques and analyses that require data to be in a “tall” structure.

For a detailed overview of how to use and correctly set the parameters for each function, consult the documentation created for the package, available online.

Runtime execution errors are handled by displaying the error description returned natively by the web service in the Python console, along with custom error messages if incorrect arguments are passed to the functions in the various modules.

## Results

### Installation

*pyBioPortal* has been published on the Python Package Index (PyPI) (https://pypi.org/project/pybioportal/) and on Anaconda.org (https://anaconda.org/matteo.valerio/pybioportal), the 2 most popular platforms used for distributing Python packages.

The installation of the package is straightforward and follows the standard procedure for both platforms with the following commands:


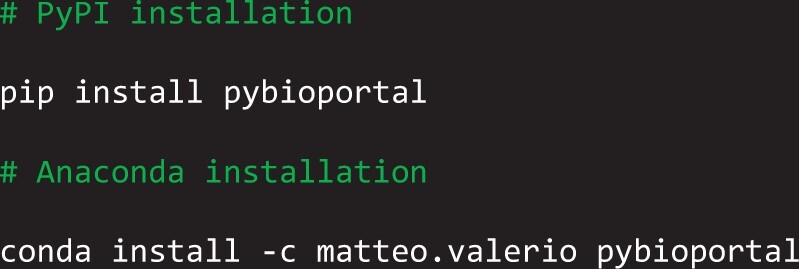


### Example of usage

*pyBioPortal* implements a simple and efficient interface between the user and the cBioPortal database. To illustrate its usage, an example is provided for retrieving clinical data on patient survival from a study available in cBioPortal. The study considered is related to The Cancer Genome Atlas Serous Ovarian Cancer project,^11^ identified by the code “ov_tcga_pub.”

First, import the module that handles the specific type of data, in this case, “clinical_data.” Next, identify the appropriate function from the module for the data to be retrieved, in this case, “fetch_all_clinical_data_in_study.” Then, specify the variables to be acquired, according to the cBioPortal documentation. In this example, the selected variables are “os_status” (overall survival status), “os_months” (overall survival time in months), and “platinum_status” (the patient’s response to platinum-based treatment). Finally, based on the specifications of the parameters to be passed to the function, as reported in the *pyBioPortal* documentation, it can be executed as shown in the code below:


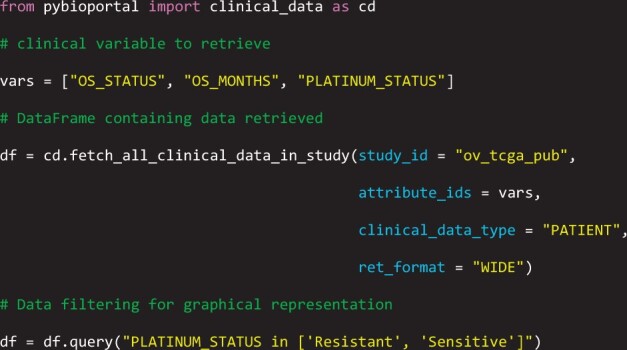


**Figure 2.**
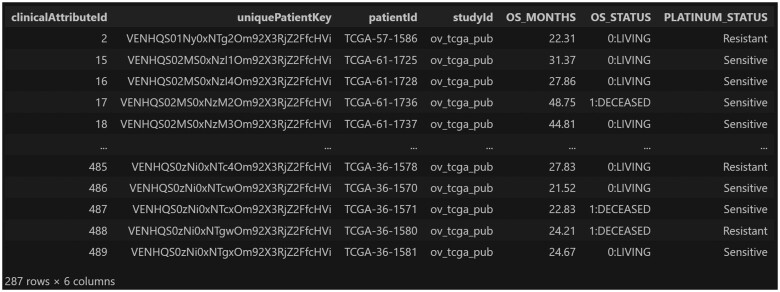
Pandas DataFrame returned by the function execution.

The Pandas DataFrame returned by the function is shown in Figure 2.

**Figure 3.**
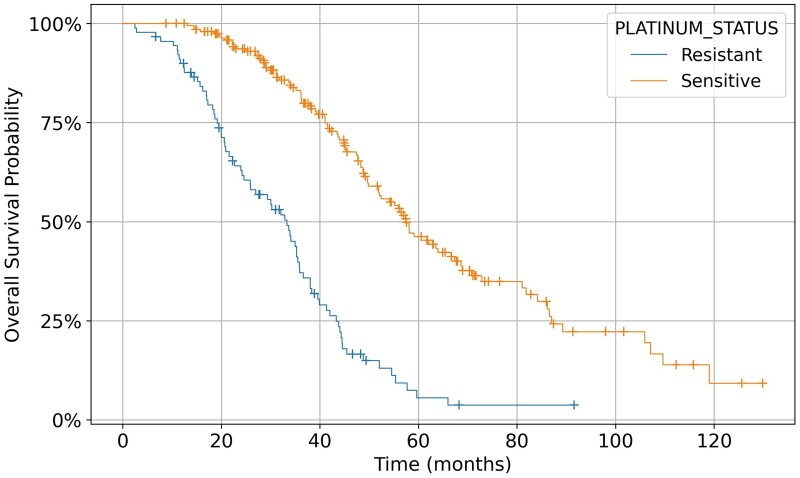
Kaplan-Meier survival curves obtained using Lifelines package with the clinical data retrieved from cBioPortal.

In this example, an additional argument, “ret_format,” is passed to the function “fetch_all_clinical_data_in_study,” specifying that the DataFrame should be returned in “wide” format. This way, each row of the DataFrame corresponds to a patient, making it very convenient to use, for example, to plot Kaplan-Meier survival curves using the Python package Lifelines,^12^ obtaining the graph shown in Figure 3.

For further details on how to use the functions of the various *pyBioPortal* modules, refer to the online documentation published on Read the Docs (https://pybioportal.readthedocs.io), which includes various application examples organized in Jupyter Notebooks.^13^

### Configuration for local instances of cBioPortal

The *pyBioPortal* package is designed to interact with the public API of cBioPortal, with the default Uniform Resource Locator (URL) set to https://www.cbioportal.org/api. However, the package also supports the use of local instances of cBioPortal, which can be implemented by institutions or companies that wish to maintain their data on private servers.

To use a local instance instead of the public one, API base URL can be configured using the “configure_base_url” function from the *pyBioPortal* configuration module (“__config”). This allows HTTP requests to be sent to a specific cBioPortal instance, installed locally or on a private server, rather than to the public instance. The following code example shows how to set a custom base URL:


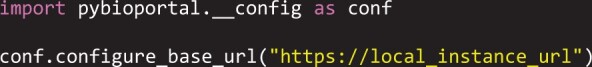


This feature is particularly useful for users working with sensitive data or who require complete control over their analysis environment, avoiding dependence on the public cBioPortal infrastructure.

## Discussion

The *pyBioPortal* package was developed with the main goal of simplifying interaction with the public cBioPortal API, making biological and clinical data more accessible to researchers or university students who are not familiar with advanced programming.

This tool significantly reduces the complexity of the data acquisition process, allowing users to focus on analysis rather than data extraction techniques. As demonstrated in the results, data are acquired with just a few lines of Python code, a simplified approach that enhances efficiency and contributes to improving the reproducibility of analyses, a key objective in bioinformatics research.

From the official documentation of cBioPortal (https://docs.cbioportal.org/web-api-and-clients/), there are 2 main ways to access the API using Python: the Bravado package, which allows direct interaction with the API, and the cbio_py package, a wrapper for the API.

Compared to these approaches, *pyBioPortal* provides advantages in terms of usability and functionality. While Bravado allows direct and flexible interaction with the API, it requires familiarity with API specifications. The retrieved data are structured as complex objects in the native API format, often requiring significant post-processing before data analysis. The cbio_py package simplifies this process by returning the retrieved data as a list of dictionaries, as well as providing it in the native format. However, as reported in the documentation, cbio_py is limited to GET endpoints and does not handle treatment-related data. Additionally, it still requires post-processing to prepare data for analysis. In contrast, *pyBioPortal* handles the entire set of cBioPortal API endpoints, including both GET and POST methods, and provides data directly in the user-friendly Pandas DataFrame format. This allows flexible and comprehensive access to various types of clinical and genomic data, facilitating data analysis.

The ability to easily configure queries through function arguments and receive data in Pandas DataFrame format provides a significant advantage to users, who can leverage useful functions applicable to DataFrames for describing and presenting data.

Currently, *pyBioPortal* uses the Pandas library to convert data obtained from cBioPortal into DataFrames, providing a familiar and versatile format for data analysis in Python. However, we recognize that processing very large datasets may present memory and performance limitations. Additionally, the retrieval time of large datasets from the cBioPortal API could be lengthy, slowing down the execution of code that depends on it. In the future, implementing techniques such as data streaming or data chunking could improve the handling of large datasets. These approaches would allow loading and processing data in smaller portions, reducing memory usage and increasing the efficiency of the package on extensive datasets. To optimize both memory usage and API response times, users are encouraged to specify targeted queries to avoid loading unnecessary data for their analysis.

The use of the Pandas DataFrame format, popular within the scientific community, enables integration of this information into custom Python scripts and pipelines for further processing and analysis, as well as leveraging Jupyter Notebooks to support workflows in scientific computing.

Given that cBioPortal is adopted by numerous institutions and companies with local installations, *pyBioPortal* includes functionality to configure the server URL for API interactions. This allows users to send requests to private servers, keeping data internally and adapting to the needs of managing sensitive data that require complete control over the analysis environment.

In the future, it is planned to implement new supporting features that will enhance and improve usage, such as user authorization support for local cBioPortal instances and the development of built-in analysis functions. Moreover, future API updates will be considered and included in subsequent versions of the package.

## Conclusion

In this paper, we presented *pyBioPortal*, a Python package useful for retrieving data from cBioPortal through its REST web services, which provides quick and easy access to heterogeneous biological and clinical data.

The aim is to provide a significant contribution to the bioinformatics community by offering a tool that facilitates programmatic access to cBioPortal data and enhances the reproducibility of the analyses in cancer research. For this reason, *pyBioPortal* has been distributed on GitHub and released as an open-source project under the Berkeley Software Distribution version 3 (BSD-3) license to maximize accessibility, collaboration, and usage.

## Data Availability

*pyBioPortal* is freely available on PyPI at https://pypi.org/project/pybioportal/ and on Anaconda.org at https://anaconda.org/matteo.valerio/pybioportal. Documentation and examples are available online at https://pybioportal.readthedocs.io. *pyBioPortal* source code is publicly accessible at the following GitHub repository: https://github.com/Matteo-Valerio/pyBioPortal.
